# Remuneration of donors for cell and gene therapies: an update on the principles and perspective of the World Marrow Donor Association

**DOI:** 10.1038/s41409-024-02246-x

**Published:** 2024-02-23

**Authors:** Lina Hamad, Salmah Mahmood Ahmed, Eefke van Eerden, Suzanna M. van Walraven, World Marrow Donor Association Cellular Therapy Committee, Laura Machin

**Affiliations:** 1 Lancaster Medical School, Lancaster University, Lancaster, UK; 2 Anthony Nolan, London, UK; 3 World Marrow Donor Association, Leiden, The Netherlands; 4 Sanquin Blood Supply, Amsterdam, The Netherlands; 5 Imperial College London, London, UK

**Keywords:** Stem-cell research, Medical ethics, Haematopoietic stem cells

## Abstract

The cell and gene therapy (CGT) sector has witnessed significant advancement over the past decade, the inception of advanced therapy medicinal products (ATMPs) being one of the most transformational. ATMPs treat serious medical conditions, in some cases providing curative therapy for seriously ill patients. There is interest in pivoting the ATMP development from autologous based treatments to allogenic, to offer faster and greater patient access that should ultimately reduce treatment costs. Consequently, starting material from allogenic donors is required, igniting ethical issues associated with financial gains and donor remuneration within CGT. The World Marrow Donor Association (WMDA) established the Cellular Therapy Committee to identify the role WMDA can play in safeguarding donors and patients in the CGT field. Here we review key ethical principles in relation to donating cellular material for the CGT field. We present the updated statement from WMDA on donor remuneration, which supports non-remuneration as the best way to ensure the safety and well-being of donors and patients alike. This is in line with the fundamental objective of the WMDA to maintain the health and safety of volunteer donors while ensuring high-quality stem cell products are available for all patients. We acknowledge that the CGT field is evolving at a rapid pace and there will be a need to review this position as new practices and applications come to pass.

## Background

The field of cell and gene therapy (CGT) witnessed substantial progress over the past decade, leading to the approval of over forty CGT products in different markets across the world [1]. CGT products include a vast range of innovative therapies of varying complexity. Cell-based therapies encompass somatic cell therapies, stem cell lines, tissue engineered products and other types of cells and tissues used for therapeutic indications [2–4]. Gene therapy alters the expression of a certain gene, or changes the genetic properties of cells [2, 3]. Advanced therapy medicinal products (ATMPs) are cell-based or tissue-based therapies whose processing necessitates manipulation, resulting in an alteration of the biological properties of these cells or tissues [2, 3]. Globally, there is a major pipeline set in place to accelerate the development of these products. In the United States, Europe, and Asia, expedited programs are available for sponsors to fast-track regulatory approval for ATMPs treating serious and orphan conditions [5–8].

Developments of CGT products initially targeted autologous applications aimed at treating oncological and haematological diseases [9, 10]. Recently, interest in allogeneic therapies peaked, reflected by a 33% increase in allogeneic developments in 2022 compared to the previous year [11]. Developed using donor cells as uniform starting material, allogeneic sources offer access to faster “off-the-shelf” products that can be used in multiple recipients, result in more predictable manufacturing and performance, decrease production costs, and ultimately increase patient access [12, 13]. As such, there is increased reliance by the CGT industry on various donor graft sources including cord blood, hematopoietic stem cells (HSCs) and other marrow-derived cellular materials like mononuclear cells (MNCs), mesenchymal stem cells (MSCs) and T-cells (hereinafter referred to as cellular materials).

One of the fundamental objectives of the World Marrow Donor Association (WMDA) is to maintain the health and safety of volunteer donors while ensuring high-quality stem cell products are available for all patients. In light of the remarkable advances in CGT and the increased dependence on donor stem cell products for the development of CGT globally, the WMDA established the Cellular Therapy Committee to identify the role WMDA can play in safeguarding donors and patients in the CGT field. Recently, provision concerns within both the transplantation and CGT communities have been raised with regards to how donor stem cells can be sought, and a pipeline sustained for understanding around CGT to advance without compromising the associated donation system for patient hematopoietic cell transplantation (HCT). In addition, reliance on donor cells as starting materials for CGT development presents new ethical dilemmas as the opportunity of financial gain becomes available for third parties using donor cells [14]. Whilst an important issue in the context of CGT development, the prizing of ATMPs is beyond the scope of this article for two reasons. Firstly, WMDA has no role in the pricing strategies for these therapies and, secondly, there is a lack of transparent information available on the pricing strategies agreed between the pharmaceutical company and the healthcare sectors for approved commercial use.

In view of these issues, the topic of donor remuneration has gained traction once again. WMDA promotes the importance of providing safe, high-quality, and ethically sourced donor stem cells to streamline CGT development and advance public health. At the same time, WMDA recognizes CGT is an evolving field and pressure to adapt can result in shifts in practice proceeding official regulatory guidance. WMDA previously issued a statement on donor remuneration, albeit primarily related to immediate, direct patient need as opposed to circumstances in which there is no direct patient need [15]. Accordingly, the WMDA Cellular Therapy Committee reviewed the question on donor remuneration to arrive at an updated statement that aids in the advancement of CGT globally. For the purposes of this paper, discussions on remuneration will focus on HSCs and other marrow-derived cellular materials. WMDA acknowledges the role of cord blood in CGT is critical, however, due to the unique situation around the donation and collection of cord blood, this will be out of scope for this paper.

## Payment terminology

**Fig. 1:**
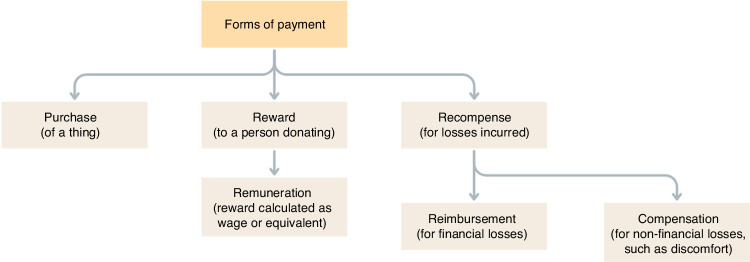
Definitions of Different Forms of Payment. Amended to illustrate payment terms. Adapted from Nuffield Council on Bioethics [16]).

The Nuffield Council on Bioethics, a UK-based independent charitable body that investigates and reports on ethical issues raised by advancements in biology and medicine, defined the following terms in relation to payments made for Substances of Human Origin (SoHO) (Fig. 1) [16]:

## Overview of the global regulatory environment in cell and gene therapy

The manufacturing of CGT products poses complex logistical challenges and is subject to global policies and regulations of variable, and sometimes, ambiguous nature [17]. Similarly, donor compensation guidelines are heterogenous, and practices vary worldwide. In the United States, the United States Food and Drug Administration (FDA) is the authority responsible for regulation of human cells, tissue, and cellular and tissue-based products [18]. In 2011, the decision made by the US Court of Appeals for the 9^th^ Circuit made donor remuneration for peripheral blood stem cell collection (PBSC) legal in states within the Ninth Circuit [19]. This decision was followed by a heated debate in the medical and legal communities, with advocates arguing for payment as a necessary step to increase donations, while opponents believed the decision to be unethical, leading to exploitation of vulnerable populations [20]. The Department of Health and Human Services (HHS) initially filed an appeal against the decision. Months later, however, the HHS appeal was withdrawn, bringing the 9th Circuit Court’s decision back into effect [20].

In Europe, the European Union Tissues and Cells Directive (EUTCD) (2004/23/EC) regulates the procurement and testing of tissues and cells intended for human use, and cells and tissues regulated as ATMPs [21, 22]. The current directive encourages Members States to ensure voluntary and unpaid donation for human transplantation and allows compensation for expenses and inconveniences incurred as a result of donation for human transplantation, donation for research falls out of this scope. In such cases, the responsibility of determining the amount and type of compensation is either tasked to national governments or entrusted to operators directly [10, 23]. It is noteworthy to mention that a new draft regulation on standards of quality and safety for SoHO has been published by the European Commission to replace the current directive [24]. The new draft regulation plans to extend new protective measures to donors driven by voluntary and unpaid donations, however this is still currently under discussion [25]. Likewise, the United Kingdom prohibits the commercial trading of tissues and cells for human transplantation as the EUTCD is transposed into UK law, with the Human Tissue Authority (HTA) as the governing body [4, 26]. There are organisations who do market and sell donor material for research and for use in cellular therapies, this current use of donor material is out scope of the regulations [27, 28]. The Asian perspective on donor remuneration is more rigorous to that of Europe and the UK. For example, the Human Biomedical Research Act (HBRA) in Singapore prohibits commercial trading of human tissue for use in research, therapy or any other purpose and any advertisements of such trading [29].

## Inappropriate compensation

**Fig. 2:**
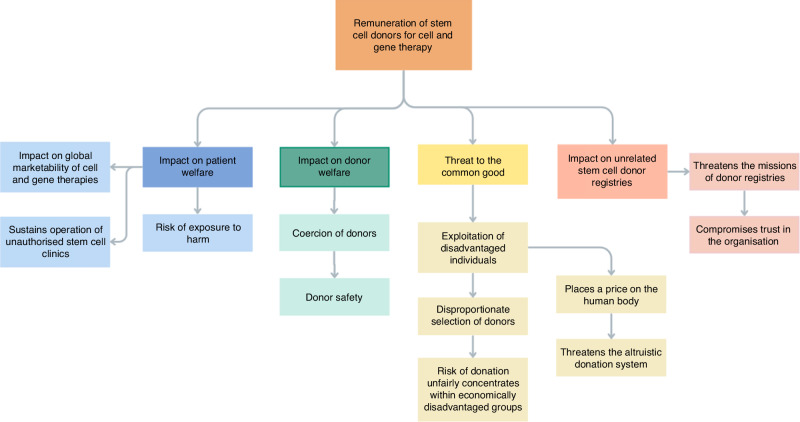
Overview of Ethical Concerns Related to Remuneration of Unrelated Donors for Cell and Gene Therapy Research and Development.

Donor reimbursement is founded upon the premise that no financial incentive or disincentive should influence a person’s decision to become a donor, making the removal of disincentives such as lost wages and care expenses permissible [30, 31]. It is common practice for unrelated stem cell donor registries (DRs) to recompense donors for travel expenses, subsistence, and loss of earnings due to the donation process when sufficient evidence is available [15]. In this context, a robust stratified claims assessment procedure is required before compensation is issued to accurately assess claims across the range of costs. These practices are not considered remuneration for the purpose of this discussion. However, an amount of compensation that is large enough to persuade potential donors to consent against their better judgment is an unacceptable form of compensation [23]. In that regard, some compensation practices by select procurement organizations supplying donor cells for CGT constitute a financial incentive with the potential to influences donors’ decisions to donate. Examples of such practices include online advertisements offering potential stem cell donors’ monetary compensation for attending an initial screening appointment, advertisements on social media offering repeated financial rewards for referring others to donate, and compensation offers that go well and beyond the losses incurred [32–34]. According to the Nuffield Council on Bioethics, to ascertain whether a particular non-altruist-focused intervention is harmful, the welfare of donors, the welfare of other closely concerned individuals (in this case, patients), the potential threat to the common good, and the professional responsibilities of individuals and organizations involved should all be closely scrutinized [16]. In this paper, we discuss how remunerating volunteer donors of HSCs and other marrow-derived cellular materials for CGT research and development has a negative impact on all four elements in question and remains detrimental to both the clinical transplantation community and the CGT community (Fig. 2).

## Welfare of donors

The decision to donate SoHO should be arrived at without any pressure or undue inducement for it to be considered voluntary [35, 36]. This principle is imperative as the act of donation entails subjecting a donor to a medical procedure for which no direct benefit can be derived. Although non-stimulated collection is lower in risk than mobilized peripheral blood stem cell collection (PBSC) using stimulating medications, both methods can result in harmful side effects which should be reported via the Serious (Product) Events and Adverse Reactions (S(P)EAR) reporting tool [37, 38]. Informed volunteer donors, nonetheless, consent to this procedure knowing this risk will not be offset by any consequent personal benefits. The introduction of financial incentives places donors’ safety at risk as some donors may be driven to assume the short-term financial benefits outweigh the risks associated with the donation procedure. Subsequently, some donors may reluctantly consent to donate solely based on the possibility of financial reward. When the amount of compensation for stem cell donation becomes proportional to the level of risk donors agree to, concerns over undue inducement intensify.

It is imperative to recognise that the role of stem cell donors in CGT is evolving and any subsequent implications are likely to clarify over time and experience. Regardless of the purpose of donation, however, respect for human dignity should always govern donation practices to ensure the intrinsic value of the human body remains protected. In that regard, multiple appeals can be found in the literature for the establishment of longitudinal governance structures between procurement organizations and donors of SoHO that go beyond informed consent [39]. These appeals are based on concerns over the ability of consent as a tool to adequately protect the dignity of donors, particularly when there is potential for financial gain by third parties using donor cells [14]. Remunerating donors could exacerbate these concerns as the potential for undue inducement deepens amid increasing international concerns over the commodification of SoHO [40]. Non-remuneration, therefore, remains the best approach to advance the field of CGT while ensuring respect for the fundamental principle of human dignity.

## Welfare of patients

Harm to patients as a result of donor remuneration was extensively discussed in the previous statement and the premise of that discussion remains valid here as well. The possibility of remuneration may prompt potential donors to withhold information that can result in their deferral for fear of missing out on financial reward [15]. An intervention that has the potential to jeopardize the screening and evaluation process of donors may risk transmission of diseases from the donor to the recipient. This can have detrimental effects on patients, especially in the context of CGT, where therapies developed using a single donor have the potential to be used in the treatment of multiple recipients [12]. While global regulations on quality control and safety of ATMPs under development exist, and robust screening mechanisms are rapidly advancing, the risks imposed on patients by a remunerating system cannot be fully eliminated.

Furthermore, there is a significant body of literature on unproven stem cell-based interventions and the proliferation of unregulated stem cell clinics offering patients unauthorized cell therapies [41–43]. Initially considered a public health problem constricted to countries with insufficient regulatory oversight, this trend has now been observed worldwide, including the USA and Europe [44–46]. Reports of patients suffering from serious and sometimes fatal side effects following the use of unproven and unregulated cell-based therapies exist [45], and while most businesses were reported to have been marketing autologous cell-based interventions, some allogeneic interventions have also been reported [43]. Donor remuneration could indirectly sustain the operation of these clinics and increase access to unapproved therapies, causing more harm to patients.

## Potential threat to the common good

Remuneration advocates may argue that donation for CGT might not carry the same altruistic sentiment as donation for direct patient treatment. Monetary incentives could, therefore, encourage more individuals to donate for CGT. Currently, there is no evidence to support the notion that donors are less likely to donate for CGT compared to direct patient treatment. Although studies on the effect of financial rewards in incentivising donations of other SoHO demonstrate inconsistent results across different populations [47–49], preliminary evidence in Canada and the UK suggests an overwhelming willingness among registered prospective donors to voluntarily donate stem cells and other types of tissues for CGT [50, 51]. Participants viewed donations for CGT as an opportunity for them to benefit the wider good by helping multiple recipients as opposed to one [52].

Remuneration or fixed rate-compensation where permissible and culturally acceptable, can be seen in other donation settings such as plasma donation or donation of small blood volumes. Although this practice does not seem to cause potential harm to donor safety and welfare, there is insufficient evidence to assess its impact on the quality of the blood provided [53]. Moreover, evidence suggests blood donors remain significantly committed to non‐remunerated blood donation, even when remuneration may be possible [49]. DRs have a unique asset which is a committed donor base, with whom regular contact is made through various mediums, be it social media or via email. During these contact efforts, the importance of their commitment and the link to helping patient lives is reinforced. We acknowledge current developments in the CGT field could act as another opportunity for donors to participate in helping patients, their donated material can help advance science to develop the next generation of therapies that will cure patients. At this stage, however, we do not have sufficient data to draw from a firm conclusion that an offer of remuneration will not interfere with donor commitment, and by extension, altruistic donations. This is the case for blood donation as well [47, 49, 54]. More research on donor behavior is therefore needed to explore the possible positive and negative outcomes that might result from donor remuneration.

Remuneration may also be morally problematic given its potential to attract financially disadvantaged persons. This argument was previously challenged by PBSC remuneration advocates, arguing that the low human leukocyte antigen (HLA) matching odds associated with the HCT donation system blunt the coercive nature of a paid market on financially disadvantaged individuals [20]. HLA matching in allogeneic cell therapies remains crucial to ensure the best possible outcome for patients, yet the specific uses of donor stem cells in CGT development make repeated donations from a single donor a possibility. This effectively means the coercive nature of a paid donation market cannot be entirely eliminated by low matching odds and remains a concern for CGT as it is for HCT. Moreover, a remunerating system can disproportionally select donors due to its potential to attract marginalised individuals. As a consequence, the burden of donation and its associated risks will unfairly concentrate within economically disadvantaged groups, jeopardizing the principle of justice.

## Responsibilities of organizations involved

Within the field of HCT, the chance of a donor undergoing a subsequent donation for the same recipient is approximately 5–10% [55], whereas the chance of matching with a second recipient after donation is <1% [56]. Despite these low odds, limits exist on the number of donations a single donor can make regardless of the method of collection (PBSC or bone marrow collection). DRs set these limits because they have a responsibility to protect the rights of donors and ensure their welfare and safety [36, 57–59]. However, as the demand for donor materials in CGT rises, donation requests from a single donor are also likely to increase. Donors may have to sit for longer and multiple collection sessions. This could have a negative impact on donors’ physical and mental health. Frequent donations from a single donor could consequently increase the burden of donation on donors [60]. Moreover, in the event a donor has a negative donation experience, subsequent requests may lead them into feeling coerced to participate again, placing their commitment at risk [60]. The potential for coercion is augmented when limits on the maximum number of times a donor can be recalled are not defined. A non-remunerating system continues to be the best approach to ensure donors’ safety and maintain donors’ trust in DRs when practices are constantly developing, and risks are not completely understood.

One of the fundamental objectives leading to the establishment of DRs is the facilitation of life saving transplantations via altruistic donations. DRs have a responsibility to ensure this objective is reflected in their practices. Commercialising SoHO without appropriate limits on the potential financial benefits generated from these cells could threaten the altruistic donation system and jeopardize trust in the organisation. In view of these concerns, it is essential to clarify some DRs charge slightly more margin than the cost of the donation process to cover the entire operational cost of maintaining a donor registry. The DR then reinvests to fund research and improve services and operations, which ultimately benefits donors and patients. Nevertheless, DRs have a duty towards donors to establish governance systems based on transparency. Procurement fees charged by DRs should therefore be within reasonable boundaries to ensure altruistic donations are not transformed into profit-driven enterprises [61]. This is a fundamental requirement if prospective donors are to develop the trust needed for them to consider donation to begin with. It is possible that some donors might question the integrity of the DR and its principal mission if donor remuneration is permissible, especially when transparency is absent. When the values of a DR are in question, many prospective donors might choose to back out from donation. This will be catastrophic for both the transplantation and the CGT communities.

## Impact on global marketability

A remunerating system can compromise the global marketability of CGT. As previously discussed, guidelines on donor compensation can be ambiguous and may differ considerably between countries. Inequities in global patient access to cell and gene therapies have already manifested due to the high cost of the treatments resulting in withdrawal of the treatment due to regional healthcare providers’ inability to reach payment deals with the therapy manufacturers. A worthwhile topic for further discussion but out of scope for this publication [62, 63].

CGT developers seeking marketing authorization across multiple markets are encouraged to use ethically sourced, safe, and quality-controlled starting material from nonremunerated donors. Voluntary unpaid donation remains the best approach moving forward to guarantee donor protection, ensure patients are not exposed to harm, and maintain the sustainability of healthcare systems by avoiding further inequities in access.

## Recommendations

World Marrow Donor Association (WMDA) strives for a world where access to life-saving cellular therapies for all patients is assured and donor rights and safety are protected [64]. We are proud of our efforts to ensure the rights and safety of donors are promoted and protected. The rapid pace of developments in the CGT field necessitates innovative thinking to enable progression. The approval of the first allogeneic cell therapy for use in patients is a significant milestone for the field [65]. Several additional allogeneic products requiring the donation of starting material from a donor are in the pipeline [66]. This is a remarkable achievement and highlights the potential benefits that these therapies, and the sourced donations relied upon, can bring to patients.

This publication serves as a follow-up to WMDA’s 2011 position paper on the remuneration of hematopoietic stem cell donors [15]. The development of CGT has reinstated this discussion in a different setting, as there is now the possibility of financial profit for third parties that will be using donor cells as starting material. Whilst there may be diverging views on the remuneration of donors for their contributions, WMDA remains committed at this time to advocating for the non-remuneration of volunteer donors for all types of donations, including for stem cell transplants and cell and gene therapy based on the current evidence.

We acknowledge that the issue of remuneration is complex and can depend on various factors, including cultural and societal norms. However, WMDA Cellular Therapy Committee has provided recommendations based on expert views that support non-remuneration as the best way to ensure the safety and well-being of donors and patients alike. We recognize that the supporting regulations and guidance for cell and gene therapies are constantly evolving, and we will review our recommendations as the field advances and practices develop. Nevertheless, we believe that to achieve our goal of advancing the field while ensuring the protection of donors’ rights and well-being, the safety of patients, non-remunerated donation is the way forward for now, for stem cell and cell and gene therapy.
